# Endothelial dysfunction and myocardial injury after major emergency abdominal surgery: a prospective cohort study

**DOI:** 10.1186/s12871-020-00977-0

**Published:** 2020-03-16

**Authors:** Sarah Ekeloef, Jakob Ohm Oreskov, Andreas Falkenberg, Jakob Burcharth, Anne Marie V. Schou-Pedersen, Jens Lykkesfeldt, Ismail Gögenur

**Affiliations:** 1 Center for Surgical Science, Department of Surgery, Zealand University Hospital, Lykkebækvej 1, 4600 Koege, Denmark; 2 Faculty of Health & Medical Sciences, University of Copenhagen, 1870 Frederiksberg, Denmark

**Keywords:** Perioperative medicine, Endothelial function, Myocardial injury, Abdominal surgery

## Abstract

**Background:**

Preoperative endothelial dysfunction is a predictor of myocardial injury and major adverse cardiac events. Non-cardiac surgery is known to induce acute endothelial changes. The aim of this explorative cohort study was to assess the extent of systemic endothelial dysfunction after major emergency abdominal surgery and the potential association with postoperative myocardial injury.

**Methods:**

Patients undergoing major emergency abdominal surgery were included in this prospective cohort study. The primary outcome was the change in endothelial function expressed as the reactive hyperemia index from 4-24 h after surgery until postoperative day 3–5. The reactive hyperemia index was assessed by non-invasive digital pulse tonometry. Secondary outcomes included changes in biomarkers of nitric oxide metabolism and bioavailability. All assessments were performed at the two separate time points in the postoperative period. Clinical outcomes included myocardial injury within the third postoperative day and major adverse cardiovascular events within 30 days of surgery.

**Results:**

Between October 2016 and June 2017, 83 patients were included. The first assessment of the endothelial function, 4–24 h, was performed 15.8 (SD 6.9) hours after surgery and the second assessment, postoperative day 3–5, was performed 83.7 (SD 19.8) hours after surgery. The reactive hyperemia index was suppressed early after surgery and did not increase significantly; 1.64 (95% CI 1.52–177) at 4–24 h after surgery vs. 1.75 (95% CI 1.63–1.89) at postoperative day 3–5, *p* = 0.34. The L-arginine/ADMA ratio, expressing the nitric oxide production, was reduced in the perioperative period and correlated significantly with the reactive hyperemia index. A total of 16 patients (19.3%) had a major adverse cardiovascular event, of which 11 patients (13.3%) had myocardial injury. The L-arginine/ADMA ratio was significantly decreased at 4–24 h after surgery in patients suffering myocardial injury.

**Conclusion:**

This explorative pathophysiological study showed that acute systemic endothelial dysfunction was present early after major emergency abdominal surgery and remained unchanged until day 3–5 after the procedure. Early postoperative disturbances in the nitric oxide bioavailability might add to the pathogenesis of myocardial injury. This pathophysiological link should be confirmed in larger studies.

**Trial registration:**

clinicaltrials.gov no. NCT03010969.

## Background

The endothelium is important in the regulation of vascular homeostasis, vascular tone and influences the systemic coagulability and inflammation [1–3]. The function of the endothelial cells is affected by inflammatory and oxidative changes in the cellular environment, which can induce endothelial dysfunction [1]. Nitric oxide (NO) is an essential endothelium-derived vasodilatory substance primarily produced by the endothelial NO synthase. Endothelial dysfunction is largely characterized by an impaired endothelium-dependent vasodilation [4]. The function of the endothelium can be assessed by non-invasive digital pulse tonometry with which the nitric oxide dependent vasodilator function of the microcirculation is digitally assessed [5]. The presence of endothelial dysfunction has been associated with an increased risk of developing cardiovascular diseases [6] and the assessment of endothelial function prior to surgery might improve the risk stratification for postoperative myocardial injury and major adverse cardiac events [7]. The surgical trauma induces systemic and local inflammation, oxidative stress and changes in neuro-humoral activity [8]. Postoperative endothelial dysfunction might therefore be a consequence of the surgical stress response. The aim of this explorative prospective cohort study was to examine the early acute endothelial dysfunction and NO bioavailability after major emergency abdominal surgery, and study the potential association between endothelial function and postoperative myocardial injury and major adverse cardiovascular events.

## Methods

### Study design, setting and participants

All included patients gave their oral and written informed consent. The study was approved by the Regional Ethics Committee in Region Zealand (SJ-527) and the Danish Data Protection Agency (REG-20-2016). The study was registered with clinicaltrials.gov (No. NCT03010969) and reported according to the Strengthening the Reporting of Observational Studies in Epidemiology (STROBE) Statement. The study was an explorative prospective cohort study including patients undergoing major emergency abdominal surgery in the Department of Surgery, Zealand University Hospital, Denmark. Eligible patients were screened and included within 4–24 h of the emergent surgical procedure between October 2016 and June 2017. Eligible patients were adults (≥18 years) that underwent emergency surgery within 72 h of the admission to the Department of Surgery or an emergency reoperation. Surgical procedures included open, laparoscopic, or laparoscopically assisted procedures involving the stomach, small or large bowel, or rectum for conditions such as perforation, ischemia, abdominal abscess, bleeding or obstruction, washout/evacuation of intra-peritoneal hematoma or abscess, laparotomy/laparoscopy with inoperable pathology (e.g. peritoneal/hepatic metastases), adhesiolysis, fascial dehiscence or any reoperation meeting the criteria above. Patients were excluded if transferred directly from the operating room or the post-anesthesia care room to the intensive care unit. The study protocol did not define clinical criteria for immediate postoperative intensive care unit transfer. The transfer was done at the discretion of the attending anesthesiologist and surgeon. Research personnel prospectively collected patient data from the electronic medical records. During hospitalization, patients reported directly to the research personnel. Patients were followed-up for 30 days after surgery. Preoperative comorbidity and performance were rated with the American Society of Anesthesiologists physical status classification (ASA) [9], the revised cardiac risk score [10] and the World Health Organization performance status [11]. The attending anesthetist decided on the anesthetic regime.

### Outcomes

The primary outcome was the change in endothelial function, expressed as the reactive hyperemia index (RHI), assessed by non-invasive digital pulse tonometry. The endothelial function was assessed within 4–24 h of surgery and repeated once again between postoperative day 3 and 5. Secondary outcomes included changes in biomarkers of NO metabolism and bioavailability (plasma L-arginine, plasma asymmetric dimethyl arginine (ADMA) and plasma biopterins (dihydrobiopterin (BH_2_), tetrahydrobiopterin (BH_4_), BH_2_ and biopterin metabolites). The NO biomarkers were assessed in relation to each endothelial function measurement with non-invasive digital pulse tonometry. Clinical outcomes at 30-days after surgery included myocardial injury (peak plasma cardiac troponin I ≥ 45 ng.l^− 1^ (99th percentile URL, 10% CV at 40 ng.l^− 1^)) and the composite major adverse cardiovascular events (myocardial injury, acute coronary syndrome, congestive heart failure, stroke, cardiovascular death or sudden unexpected death, non-fatal cardiac arrest, new clinically important cardiac arrhythmia and coronary revascularization procedure). A clinically important cardiac arrhythmia was defined as an event that led to a medical or procedural intervention. Cardiac troponin-I was assessed daily in the morning on postoperative day 1 to 3.

### Data sources and measurements

The measurement of the endothelial function was performed with an EndoPAT2000 (Itamar medical ltd, Israel). The EndoPAT assessment, a non-invasive digital pulse tonometry, has been described elsewhere [12]. In short, the digital pulse amplitude is assessed at rest and during reactive hyperemia. The assessment has three phases: baseline (resting phase), occlusion and hyperemia. A finger-probe was placed on each index finger. One serving as a control in order to adjust for any systemic effects. The assessment was initiated with the baseline assessment of the finger pulse amplitude (baseline phase). After 5 min, a blood pressure cuff on the upper arm was inflated to 200 mmHg (suprasystolic) and the blood flow to the forearm was ceased (occlusion phase). After 5 min of forearm ischemia, the cuff was deflated and the finger-pulse amplitude was automatically assessed for 5 min during reperfusion of the forearm (hyperemia phase). The endothelial function was expressed as RHI, which was calculated automatically. RHI is defined as the ratio between the finger-pulse amplitude during hyperemia and rest (baseline) [13].

Blood was drawn into 4 ml EDTA tubes for analysis of NO biomarkers. For analysis of L-arginine and ADMA, blood samples were centrifuged for 10 min at 3000 g and plasma was snap-frozen and stored at − 80 °C. Quantification of L-arginine and ADMA was achieved by HPLC with fluorescence detection as described by Schou-Pedersen et al. [14] For analysis of biopterins, blood was mixed with dithioerythritol and centrifuged at 2000 g for 5 min. Plasma was snap-frozen and stored at − 80 °C. Biopterins were analyzed using HPLC with fluorescence detection as described previously [15]. In order to reduce the risk of bias, all experimental procedures were standardized and performed by trained research personnel who was unfamiliar with the patients’ clinical condition.

### Study size and statistics

The study was explorative; therefore, a predefined sample size calculation was not performed. Instead, all patients that presented for major emergency abdominal surgery were consecutively screened and included from October 2016 to June 2017. The study was a predefined substudy of the POETRY abdominal study that included patients from October 2016 to November 2018 (clinicaltrials.gov No. NCT03010969). Continuous data were expressed as mean (standard deviation or 95% confidence interval) or median (IQR) depending on data distribution. Histograms and QQ-plots of residuals were performed to describe data distribution and homogeneity of variances. The endothelial function, expressed as RHI, and level of NO biomarkers at 4–24 h vs. postoperative day 3–5 were compared with a paired t-test. A mixed model was performed to evaluate the change of RHI and NO biomarkers stratified on the presence of myocardial injury or major adverse cardiovascular events. An unstructured variance-covariance structure was chosen since this model had the smallest AIC values and − 2 Log Likelihood scores. The correlations between RHI and L-arginine, ADMA and the L-arginine/ADMA ratio were tested. The correlations were expressed with scatter-plots and Pearson correlation coefficients. Statistical analyses were considered significant if the two-sided *p*-value < 0.05. All statistical analyses were performed with SAS version 9.4 (SAS Institute A/S, U.S.).

## Results

### Study population and descriptive data

**Fig. 1:**
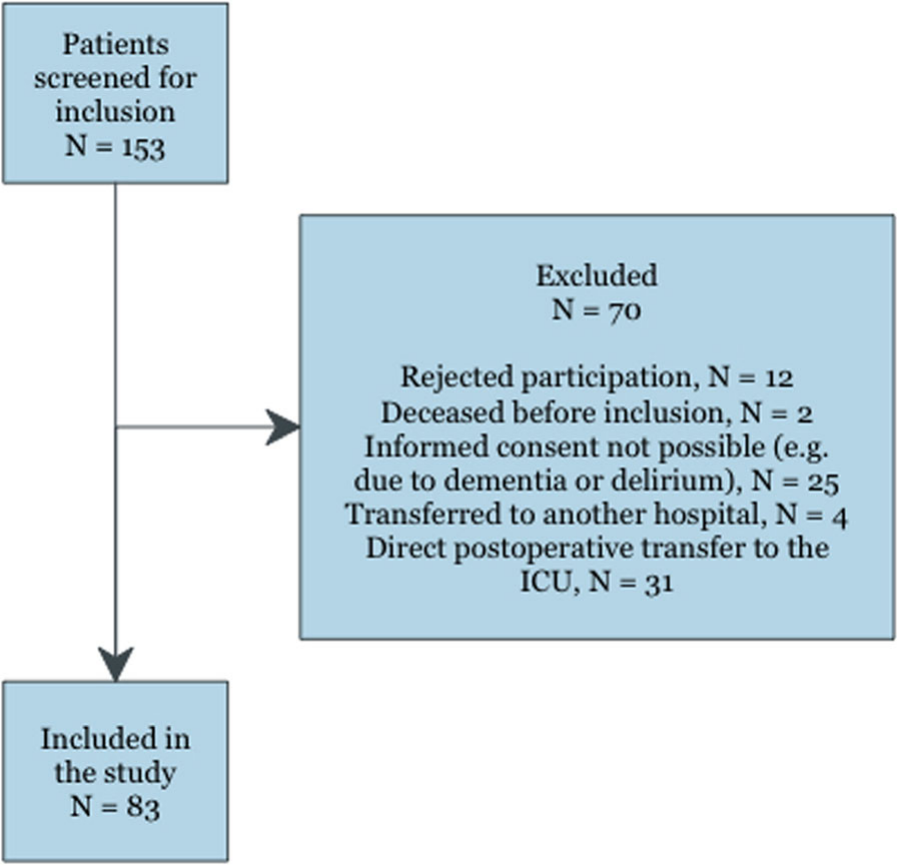
Patients flowchart

In total, 153 patients were consecutively screened for inclusion between October 2016 and June 2017. We ended up including 83 patients in this study, Fig. 1. The mean age was 64.1 (SD 15.8) years and 53% were male. The majority of the patients had a performance score of 0–1 (95.2%) and was ASA I-II (62.7%). The most common cardiovascular risk factors were hypertension (31.3%) and ischemic heart disease (8.4%). Baseline patient characteristics are available in Table 1. Total intravenous anesthesia with propofol and remifentanil was the most common anesthetic regime and 44.6% had a perioperative epidural. All but six patients suffered peroperative hypotension (systolic blood pressure < 100 mmHg for > 5 min). The surgical procedures are specified in Table 2.

**Table 1:** Basic characteristics of patients undergoing major emergency abdominal surgery

Characteristics	Whole cohort (***n*** = 83)
Age; years	64.1 (15.8)
Male	44 (53.0%)
BMI	25.3 (5.4)
Smoking (no/former/active)	35 (42.1%)/32 (38.6%)/16(19.3%)
Alcohol (none, ≤ 7 units/week, > 7 units/week)	35 (42.2%)/27 (32.5%)/21 (25.3%)
Preoperative sBP/dBP; mmHg	140.3 (22.2)/78.9 (13.0)
Hemoglobin; g.dl^−1^	13.4 (2.7)
Leucocytes; × 10^9^.l^−1^	12.6 (6.3)
CRP; mg.l^−1^ (median (IQR))	11.0 (54.0)
Thrombocytes; × 10^9^.l^−1^	283.6 (118.0)
Creatinine; mg.dl^−1^	0.93 (0.43)
Albumin; g.l^−1^	35.0 (6.9)
**Cardiovascular risk factors**	
Ischemic heart disease	7 (8.4%)
Atrial fibrillation	5 (6.0%)
Hypertension	26 (31.3%)
Congestive heart failure	3 (3.6%)
Peripheral arterial disease	6 (7.2%)
Cerebrovascular disease	5 (6.0%)
Diabetes	6 (7.2%)
RCRI (1/2/≥3)	67 (80.7%)/13 (15.7%)/3 (3.6%)
ASA (1–2/3–4)	52 (62.7%)/31 (37.3%)
Performance score (0–1/2–3)	79 (95.2%)/4 (4.8%)
**Preoperative medication**	
Acetylsalicylic acid	17 (20.5%)
Statin	14 (16.9%)
ADP-receptor inhibitors	9 (10.8%)
Anticoagulation	5 (6.0%)
Beta blockers	7 (8.4%)
Calcium channel blockers	9 (10.8%)
Diuretics	20 (24.1%)
ACE-I/ARBs	18 (21.7%)
Antidiabetics (per oral)/Insulin	4 (4.8%)/2 (2.4%)
**Assessment of endothelial function, hrs. from end of operation**	
Assessment 1 (4–24 h)	15.8 (6.9)
Assessment 2 (POD3–5)	83.7 (19.8)

Data are expressed as mean (standard deviation) or frequencies (%) unless otherwise indicated

*ACE-I* angiotensin-converting-enzym inhibitor; *ARBs* angiotensin-receptor blocker; *ASA* American Society of Anesthesiologists Classification; *BMI* Body Mass Index; *CRP* C-reactive protein; *dBP* diastolic blood pressure; *POD* postoperative day; *RCRI* Revised Cardiac Risk Index; *sBP* systolic blood pressure

**Table 2:** Peroperative surgical and anesthetic characteristics

	Whole cohort (n = 83)
**Primary surgical procedure**	
Upper gastrointestinal surgery	7 (8.4%)
Small bowel resection	15 (18.1%)
Large bowel resection	10 (12.0%)
Laparotomy (no resection)*	39 (47.0%)
Combined small and large bowel resection	4 (4.8%)
Other	8 (9.6%)
**Peritoneal contamination (none /serous/local contamination/diffuse contamination)**	53 (63.9%)/9 (10.8%)/9 (10.8%)/12 (14.5%)
**Duration of surgery; min (median (IQR))**	115.0 (72.5)
**Duration of anesthesia; min (median (IQR))**	157.0 (88)
**TIVA/volatile/combination**	45 (54.2%)/29 (34.9%)/9 (10.8%)
**Perioperative epidural**	37 (44.6%)
**Peroperative hypotension**	77 (92.8%)

*surgical finding of e.g. adhesions, abdominal abscess, inoperable pathology

Values are frequencies (proportion) unless otherwise indicated

Peroperative hypotension was defined as > 5 min with systolic blood pressure < 100 mmHg

*TIVA* total intravenous anesthesia

Sixteen out of 83 patients (19.3%) had a major adverse cardiovascular event within 30 days of surgery, the most common being myocardial injury (11/83 patients, 13.3%). Other than that, one patients had congestive heart failure (1.2%), one patient had a non-fatal cardiac arrest (1.2%), and four patients had a new clinically important cardiac arrhythmia (4.8%). In total, 29 out of 83 patients (34.9%) had a postoperative surgical complication (Clavien-Dindo ≥3) and 13 out of 83 patients (15.7%) had sepsis within 30-days of surgery. Only two patients (2.4%) had severe anemia defined as a b-hemoglobin < 6.9 g∙dL^− 1^ within three days of surgery. Median lengths of stay was 7.0 (q1-q3, 5.0–12.0) days.

### Endothelial function and nitric oxide biomarkers

**Fig. 2:**
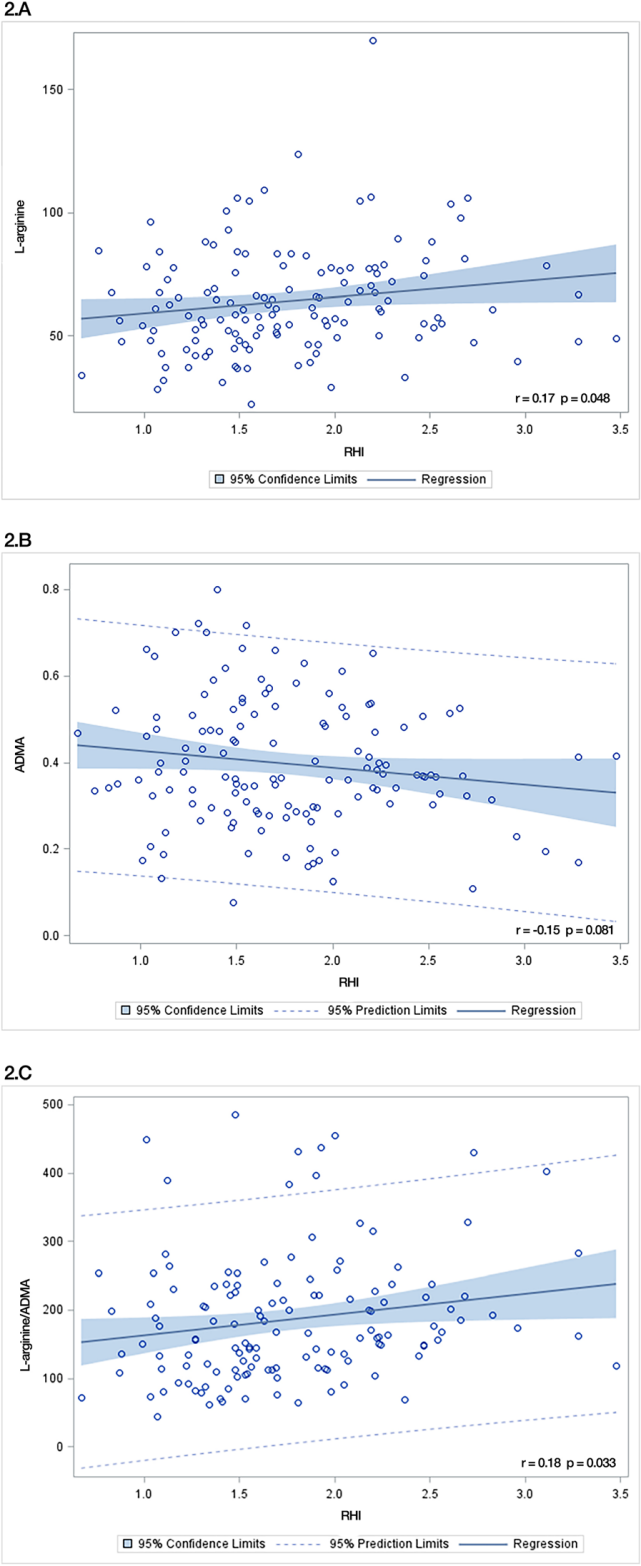
Correlations between reactive hyperemia index and nitric oxide biomarkers. A. The correlation between reactive hyperemia index (RHI) and L-arginine. B. The correlation between RHI and asymmetric dimethyl arginine (ADMA). C. The correlation between RHI and the L-arginine/ADMA ratio. r = Pearson correlation coefficient

The first assessment of the endothelial function, 4–24 h, was performed 15.8 (SD 6.9) hours after surgery and the second assessment, postoperative day 3–5, was performed 83.7 (SD 19.8) hours after surgery. RHI was 1.64 (95% CI 1.52–1.77) at 4–24 h after surgery and 1.75 (95% CI 1.63–1.89) at postoperative day 3–5, *p* = 0.34. Four patients had a reoperation between the first and second endothelial assessment. L-arginine and ADMA both increased significantly in the postoperative period resulting in an unchanged L-arginine/ADMA ratio. BH_4_ and the total biopterin level decreased significantly (*p* = 0.0008 and *p* = 0.0001, respectively), while BH_2_ and the BH_2_/BH_4_ ratio remained stable, Table 3. Positive correlations were found between RHI and L-arginine (Pearson correlation coefficient 0.17, *p* = 0.048) and between RHI and the L-arginine/ADMA ratio (Pearson correlation coefficient 0.18, *p* = 0.033), Fig. 2.

**Table 3:** Reactive hyperemia index and nitric oxide bioavailability assessed at two time points after surgery

	Assessment 1 4–24 h	Assessment 2 POD 3–5	***p*** value
**Reactive hyperemia index**	1.64 (1.52–1.77)	1.75 (1.63–1.89)	0.34
**L-arginine;** μmol**.l**^**− 1**^	56.94 (52.45–61.43)	72.76 (67.62–77.91)	< 0.0001
**ADMA;** μmol**.l**^**− 1**^	0.38 (0.34–0.41)	0.42 (0.39–0.46)	0.048
**L-arginine/ADMA**	158.46 (141.25–177.75)	175.59 (155.75–197.95)	0.097
**BH**_**4**_**; nmol.l**^**− 1**^	7.90 (7.22–8.59)	6.47 (5.69–7.25)	0.0008
**BH**_**2**_**; nmol.l**^**− 1**^	4.11 (3.67–4.61)	3.73 (3.41–4.07)	0.23
**BH**_**2**_**/BH**_**4**_	0.56 (0.48–0.65)	0.68 (0.55–0.83)	0.052
**BH**_**2**_**and biopterin metabolites; nmol.l**^**− 1**^	11.99 (11.08–12.96)	9.89 (9.06–10.78)	0.0001

*ADMA* asymmetric dimethyl arginine; *BH*_*4*_ tetrahydrobiopterin; *BH*_*2*_ dihydrobiopterin; *POD* postoperative day

Mean with 95% confidence interval. Paired t-tests were applied to compare the assessments

### Cardiovascular events and endothelial function

**Fig. 3:**
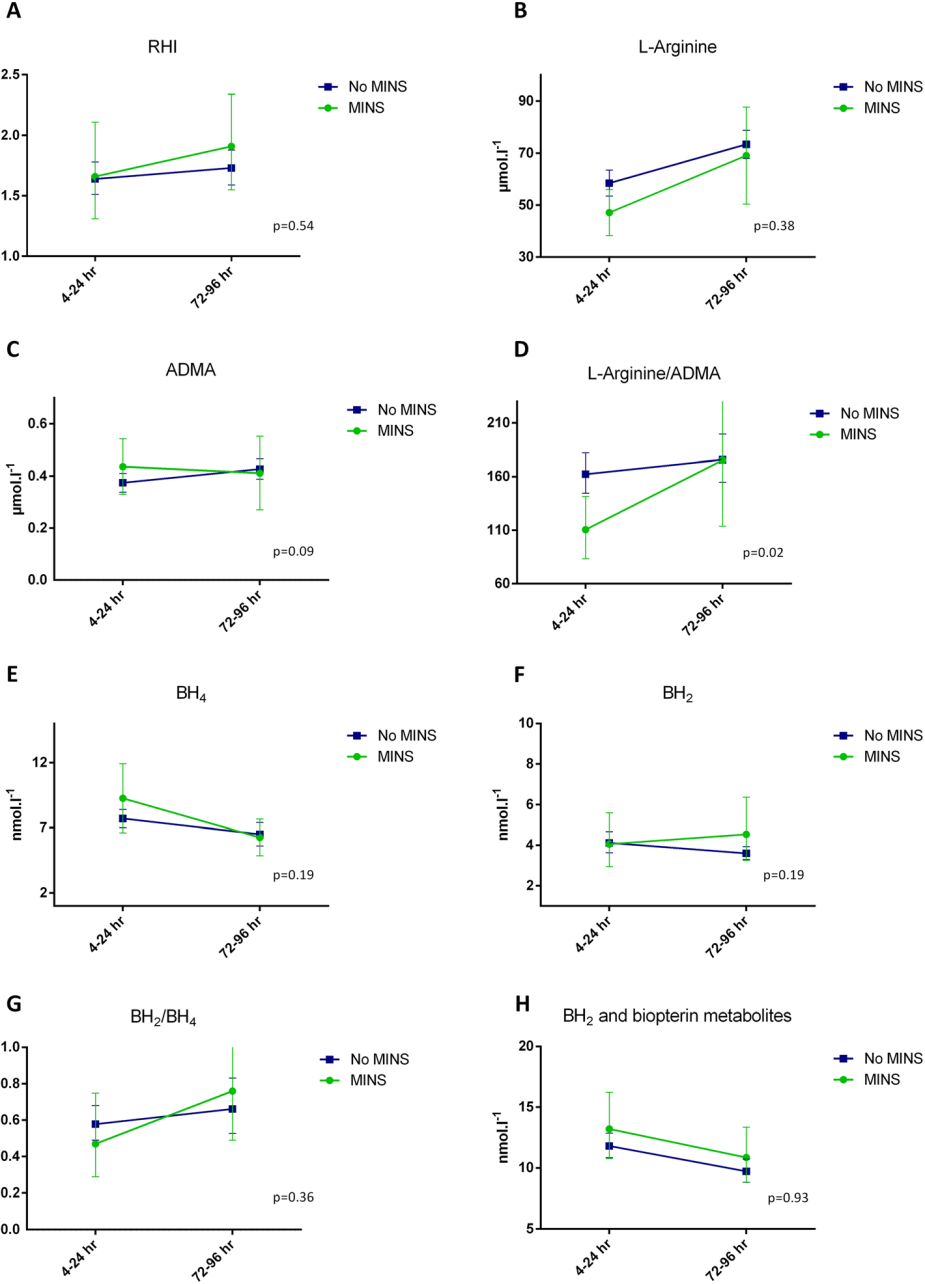
Changes in reactive hyperemia index and nitric oxide biomarkers stratified on the presence or absence of myocardial injury within the third postoperative day. Abbreviations: ADMA, asymmetric dimethyl arginine; BH_2_, dihydrobiopterin; BH_4_, tetrahydrobiopterin; MINS, myocardial injury; RHI, reactive hyperemia index

An exploratory analysis on the difference between patients with and without myocardial injury was performed, Fig. 3a-h. RHI did not differ between patients with and without myocardial injury and RHI did not change significantly over time in any of the groups, Fig. 3a. L-arginine and ADMA did not differ between patients with and without myocardial injury, but the L-arginine/ADMA ratio was significantly suppressed at 4–24 h after surgery in patients suffering myocardial injury, Fig. 3b-d.

The biopterin levels did not differ between patients with and without myocardial injury, Fig. 3e-h. No difference was observed when RHI and NO biomarkers were stratified on major adverse cardiovascular events, Table 4.

**Table 4:** Endothelial function in patients with and without major adverse cardiovascular events

	Patients suffering major adverse cardiovascular events (***n*** = 16)	Patients without major adverse cardiovascular events (***n*** = 67)	p value
**Reactive hyperemia index**			0.36
4–24 h	1.62 (1.33–1.96)	1.65 (1.52–1.80)	
POD3–5	1.88 (1.64–2.16)	1.72 (1.58–1.89)	
**L-arginine; μmol.l**^**− 1**^			0.96
4–24 h	49.54 (43.16–55.91)	58.73 (53.40–64.07)	
POD3–5	65.66 (52.57–78.76)	74.64 (68.99–80.30)	
**ADMA; μmol.l**^**− 1**^			0.24
4–24 h	0.43 (0.35–0.51)	0.37 (0.33–0.41)	
POD3–5	0.44 (0.33–0.55)	0.42 (0.38–0.46)	
**L-arginine/ADMA**			0.21
4–24 h	117.55 (95.21–145.15)	164.51 (145.79–185.62)	
POD3–5	155.04 (112.88–212.96)	181.49 (159.16–206.95)	
**BH**_**4**_**; nmol.l**^**− 1**^			0.53
4–24 h	8.75 (6.99–10.52)	7.69 (6.94–8.44)	
POD3–5	6.70 (5.47–7.92)	6.41 (5.48–7.35)	
**BH**_**2**_**; nmol.l**^**−1**^			0.92
4–24 h	4.85 (3.54–6.63)	3.95 (3.50–4.46)	
POD3–5	4.46 (3.44–5.77)	3.57 (3.26–3.90)	
**BH**_**2**_**/BH**_**4**_			0.88
4–24 h	0.59 (0.41–0.85)	0.56 (0.47–0.66)	
POD3–5	0.70 (0.49–0.99)	0.67 (0.53–0.85)	
**BH**_**2**_**and biopterin metabolites; nmol.l**^**− 1**^			0.97
4–24 h	13.74 (11.42–16.52)	11.59 (10.63–12.64)	
POD3–5	11.22 (9.65–13.05)	9.58 (8.66–10.61)	

*ADMA* asymmetric dimethyl arginine; *BH*_*4*_ tetrahydrobiopterin; *BH*_*2*_ dihydrobiopterin; *POD* postoperative day

Mean with 95% confidence interval. A mixed model was applied to evaluate the development over time stratified on major adverse cardiovascular events

## Discussion

In this explorative prospective cohort study on patients undergoing major emergency abdominal surgery, the endothelial function, expressed as the reactive hyperemia index, and the NO-production, expressed as L-arginine/ADMA ratio, remained reduced during the postoperative period.

The endothelial function was severely suppressed, however, whether this endothelial dysfunction was present already before the surgical procedure is not known. Other studies in the field do report of acute changes in the endothelial function early after moderate-major non-cardiac surgery with RHI of 1.47 (95% CI 1.08–1.98) [12] and 1.52 (± 0.28) [16] early after the procedure. Likewise, the NO production expressed as the L-arginine/ADMA ratio was reduced in our study early after surgery. This pattern has been observed in other surgical studies [12, 17] and is in line with the positive correlation identified between RHI and the L-arginine/ADMA ratio. Preoperatively, the patients in our study all suffered from an acute illness and the extent of endothelial dysfunction after the emergent surgical procedure was likely the combined product of acute illness and surgical stress.

Several clinical studies have reported of an association between systemic endothelial function and the risk of coronary disease and further cardiovascular morbidity [6, 18]. Adding the preoperative RHI value to the Revised Cardiac Risk Index has been shown to improve the risk classification for postoperative myocardial injury, which supports the pathophysiological role of endothelial dysfunction in the development of myocardial injury [7]. The L-arginine/ADMA ratio is an indirect biochemical measure of the NO-production [4]. Interestingly, we found that patients suffering myocardial injury had a significantly lower L-arginine/ADMA ratio, thus a lower NO-production than patients without myocardial injury 4–24 h after surgery. This finding might indicate a role for lower perioperative NO bioavailability in the pathogenesis of myocardial injury. This analysis was explorative and should be confirmed in a larger study. We did not find any difference in the L-arginine/ADMA ratio between patients with and without major adverse cardiovascular events within 30 days of surgery.

In the vessels, NO is produced by the endothelial NO synthase (eNOS) [19]. BH_4_ is a central eNOS cofactor and a low plasma level of BH_4_ has been associated with uncoupling of eNOS [19]. The uncoupling reduces the NO production and increases systemic oxidative stress since eNOS switches to produce reactive oxygen species such as superoxide leading to the formation of peroxinitrite [20]. In this study, BH_4_ decreased over time after major emergency abdominal surgery. The level of BH_4_ depends on de novo synthesis, redox balance and biopterin clearance [19]. Systemic redox imbalance after surgery may cause BH_4_ oxidation to BH_2_ and other oxidized biopterin metabolites [21]. However, this mechanism does not explain that BH_2_ remained unchanged and that biopterin metabolites decreased significantly. An increased biopterin clearance combined with BH_4_ oxidation and a reduced de novo synthesis might in combination explain the postoperative biopterin levels. Interestingly, the BH_2_/BH_4_ ratio remained unchanged in the postoperative period. Since binding of BH_2_ to eNOS causes an uncoupling of the NO production, the BH_2_/BH_4_ ratio has been argued to reflect the extent of eNOS uncoupling and NO production [21–24]. In patients undergoing major colon cancer surgery, the preoperative BH_2_/BH_4_ ratio was 0.53 (95% CI 0.46–1.37) [12]. The ratio is comparable to the postoperative ratio found in this study, thus, the biopterin balance seems to be preserved at least until day 3–5 after major emergency abdominal surgery.

Our study has a number of strengths and limitations. The study was a prospective cohort study with prespecified methods and outcomes. The inclusion criteria were wide in order to increase the generalizability of the study; however, this did also result in a heterogenic population with a large inter-patient variance. Besides differences in the surgical stress response, patients suffered from a variety of postoperative surgical and medical complications including sepsis and pain, which potentially could have affected the systemic endothelial function. Patients transferred directly to the intensive care unit from the operation room could not be included in the study. Therefore, the results cannot be generalized to this severely ill part of the surgical population and only the patients with the best prognosis were represented. Criteria for intensive care unit transfer were not defined in the study protocol but decided by the attending clinician. This could be a potential source of selection bias. No preoperative assessment of the endothelial function was performed due to the acute setting of the study. The endothelial function was assessed within 4–24 h after surgery and on postoperative day 3–5. Such time intervals have previously been used when assessing the endothelial function after an acute event [25]. However, the large variance could add to the heterogeneity of the study. The observational design prevents us from commenting on causality. The systemic endothelial function assessed by non-invasive digital pulse tonometry has previously been correlated with coronary microvascular endothelial function assessed invasively during coronary angiography [26]. A Non-invasive assessment of the coronary microvascular function can be performed with transthoracic Doppler echocardiography of the left anterior descending coronary artery [27]. A non-invasive assessment of the postoperative coronary microvascular function could potentially have added interesting data to our study of peripheral and systemic endothelial function.

In conclusion, systemic endothelial dysfunction was present in the perioperative period after major emergency abdominal surgery and did not significantly improve during the study period. The L-arginine/ADMA ratio was reduced early after surgery in patients with myocardial injury, which might imply that an impaired endothelial NO-production adds to the pathogenesis of myocardial injury. This hypothesis-generating finding should be confirmed in larger cohort studies.

## Data Availability

The datasets used and/or analyzed during the current study are available from the corresponding author on reasonable request.

## Abbreviations

ADMA: Asymmetric dimethyl arginine
ASA: American Society of Anesthesiologists physical status classification
BH_2_: Dihydrobiopterin
BH_4_: Tetrahydrobiopterin
eNOS: Endothelial nitric oxide synthase
NO: Nitric oxide
RHI: Reactive hyperemia index
